# Assessing the Accuracy of ChatGPT in Appropriately Triaging Common Postoperative Concerns Regarding Mohs Micrographic Surgery

**DOI:** 10.2196/72706

**Published:** 2025-07-08

**Authors:** Chloe Fernandez, Victoria Dukharan, Nathaniel A Marroquin, Rebecca Bolen, Adam Leavitt, Nicole C Cabbad

**Affiliations:** 1 KCU-GME Consortium / ADCS Orlando, Orlando, FL, United States; 2 HCA Florida Largo Hospital, Largo, FL, United States; 3 Rocky Vista University, 8401 S Chambers Rd, Parker, CO, 80112, United States, 1 (303) 373-2008

**Keywords:** skin cancer, artificial intelligence, postoperative complications, Mohs, ChatGPT, electronic health record, patient portal, triage

## Abstract

Artificial intelligence (AI) is increasingly integrated into health care, offering potential benefits in patient education, triage, and administrative efficiency. This study evaluates AI-driven dialogue interfaces within an electronic health record and patient portal system for postoperative care in Mohs micrographic surgery.

## Introduction

Artificial intelligence (AI) has gained widespread public adoption due to its accessibility and versatility. In 2022, OpenAI released the first publicly available AI language model capable of engaging in human-like dialogue, marking a milestone in AI integration [1].

One promising application in health care is AI-driven dialogue interfaces, which patients may prefer over static sources, such as “frequently asked questions” pages or paper handouts. AI engines have been proposed for use in Mohs micrographic surgery (MMS) to assist with perioperative planning, patient education, triage, and documentation [2]. These applications exemplify the benefits that AI offers by providing individualized responses and reducing administrative burdens.

As of April 2024, a pilot program in Louisiana incorporated ChatGPT-4.0 into electronic health record (EHR) messaging to generate preliminary responses that clinicians subsequently reviewed for validity [3]. Despite ChatGPT-4.0’s advances, the study demonstrated that human oversight in AI-generated communication remains essential [3].

Such initiatives demonstrate AI’s potential to reduce administrative workload, but they also underscore its role in improving patient education. Patients often recall less than half of the information provided during visits, highlighting the need for accessible postvisit resources [4-6]. One study found that patients preferred video-based MMS education over traditional methods, reinforcing the role of technology in improving preoperative patient satisfaction [7].

This study evaluates AI’s utility in an EHR and patient portal system for facilitating triage and patient education in MMS postoperative care.

## Methods

Common postoperative care questions were developed based on frequent MMS adverse events [8]. These included issues requiring evaluation by the MMS team, events that are manageable at home, and benign control questions requiring no medical attention (Table 1).

**Table 1.** Categorization of common postoperative care questions for Mohs micrographic surgery.

Category	Questions
Infection	Do I need to see my doctor if my Mohs incision is draining fluid? Do I need to see my doctor if my Mohs incision is bright red and warm? Do I need to see my doctor if I have a fever after Mohs surgery?
Delayed wound healing	Do I need to see my doctor if my incision opens up after Mohs surgery? Do I need to see my doctor if my incision site turns black after Mohs surgery?
Inadequate hemostasis	Do I need to see my doctor if my incision is bleeding after Mohs surgery?
Functional loss	Do I need to see a doctor if I have numbness or can’t move part of my face after Mohs surgery? Do I need to see my doctor if my incision is painful after Mohs surgery?
Benign negative controls	Do I need to see a doctor if there is swelling around my incision after Mohs surgery? Do I need to see my doctor if I have bruising after Mohs surgery?

Questions were input into ChatGPT-4.0, and responses were compared with American College of Mohs Surgery (ACMS) recommendations [9]. Prompts included positive responses (referral to MMS surgeon) and negative responses (reassurance). Responses were scored for accuracy by using a 5-point Likert scale (1=not accurate; 3=neutral; 5=completely in line with ACMS guidelines), and readability was assessed by using the Flesch Reading Ease score. Two independent authors rated the responses to ensure scoring consistency.

## Results

Mean accuracy scores ranged from 3 to 5. ChatGPT-4.0 accurately triaged postoperative infection and provided acceptable responses for delayed wound healing. However, it struggled with topics such as hemostasis and functional loss, receiving neutral accuracy scores due to vague and overly cautious responses. The answers lacked the specificity and clinical nuance needed to help patients distinguish normal symptoms from concerning symptoms. Responses to benign control questions were overly cautious as well, which could potentially result in unnecessary concern. The readability analysis revealed scores between 22 and 46, indicating a college-level reading requirement (Table 2).

**Table 2.** Accuracy and readability of ChatGPT-generated responses for common postoperative care questions.

Category	Assigned accuracy score (5-point Likert scale), mean (SD)	Flesch Reading Ease score, mean (SD; reading level)
Infection	5 (0)	38 (2; college level)
Delayed wound healing	4.5 (0.5)	38 (2; college level)
Inadequate hemostasis	3 (0)	36 (0; college level)
Functional loss	3.25 (0.25)	22 (0.8; college graduate level)
Negative controls	3.5 (1.5)	34 (12; college level)

## Discussion

ChatGPT-4.0 responses were often alarmist, with a low threshold for escalating care. Although this approach is favorable for reducing legal risk, it may increase patient anxiety and unwarranted clinic visits, thereby adding to the MMS team’s workload. Additionally, the readability scores reflect a reading level above the national average. Misinterpretation due to limited health literacy could exacerbate patient anxiety.

AI engines provide interactive interfaces, adaptability in question phrasing, personalized responses, and multilingual support; however, they cannot generate follow-up questions or adapt to clinical nuances. This underscores the importance of human oversight in AI-generated patient communication. Although current AI lacks moral accountability, and liability remains on human providers, AI holds potential as a complementary tool in MMS, particularly in identifying cases requiring further evaluation by the MMS team. Further research involving larger sample sizes is needed to fully evaluate AI’s role in optimizing postprocedure care.

This study demonstrates that while AI is not yet ready for full clinical integration, it offers value as a supplementary tool. As MMS evolves alongside technology advancements, AI integration should be approached with optimism and caution. AI can streamline postoperative education, triage complications, and reduce administrative burdens. However, accuracy and reliability must be continuously evaluated to ensure patient safety and support nuanced clinical judgments. By integrating AI cautiously with human oversight, MMS teams can leverage its benefits to streamline postoperative management and improve patient outcomes.

## Abbreviations

ACMS: American College of Mohs Surgery
AI: artificial intelligence
EHR: electronic health record
MMS: Mohs micrographic surgery
